# Introducing a new special section—Indigenous Science and Practice in Ecology and Evolution

**DOI:** 10.1002/ece3.11718

**Published:** 2024-07-24

**Authors:** Emily A. McKinnon, Arley F. Muth

**Affiliations:** ^1^ University of Manitoba Winnipeg Manitoba Canada; ^2^ John Wiley & Sons Ltd Hoboken NJ USA

**Keywords:** Indigenous science, traditional ecological knowledge

## Abstract

Editorial introducing a new special issue in the journal.

A fulsome understanding of ecology and evolution is not possible without contributions from Indigenous traditional and local knowledge (TEK/LEK) of ecosystems. These knowledges are inherently tied to land and culture, and their incorporation or implementation requires working with Indigenous Peoples in good ways (Reid et al., 2024). Two‐eyed seeing (*Etuaptmumk* in Mi'kmaw, conceptualized by Elder Albert Marshall, Bartlett et al., 2012), Indigenous science and community‐based approaches to understanding ecology and evolution can provide insight into challenging local‐ and global‐scale problems. Indigenous peoples globally have been sustainably managing natural resources and safeguarding biodiversity for millennia (Garnett et al., 2018), thus incorporation of Indigenous knowledges and knowledge systems into biodiversity conservation is timely and necessary (Ogar et al., 2020). Combining TEK/LEK with other data sources can identify species range shifts (e.g. Service et al., 2014), climate impacts (Gagnon et al., 2020), and insights into behaviour and habitat use (Dubos et al., 2023). A growing community of Indigenous and non‐Indigenous scientists, educators and community members recently gathered at the second Turtle Island Indigenous Science Conference (https://event.fourwaves.com/2024tiisc/pages), held in atim kâ‐mihkosit (Red Dog) Urban Reserve and Star Blanket Cree Nation, in Treaty 4 Territory (Regina, Canada), to share Indigenous Science approaches to a diversity of topics in ecology and evolution and was informative for the authors of this editorial as we launch this special section.

At *Ecology and Evolution*, we invite Indigenous researchers, scientists using Indigenous practices and approaches, and those working in good ways (Reid et al., 2024) with Indigenous communities, to submit to a new special issue and ongoing special section—*Indigenous Science and Practice in Ecology and Evolution*. We welcome papers showcasing the use of Indigenous practices and approaches, alone or alongside other approaches to answer ecological and evolutionary questions. Contributions are welcome in all spaces of the journal, from long‐term ecological and observational data, natural history information (article type: Nature Note, https://onlinelibrary.wiley.com/doi/full/10.1002/ece3.6534 and https://onlinelibrary.wiley.com/doi/full/10.1002/ece3.9215), experimental research questions, community‐engaged research, research using Indigenous science methods and approaches, and successful teaching and Indigenous community engagement projects (article type: Academic Practice in Ecology and Evolution, https://onlinelibrary.wiley.com/doi/10.1002/ece3.5).

Through this special issue and ongoing special section, we are striving to highlight the importance of Indigenous science, researchers and practices, and emphasize the inherent and scientific value of these contributions (Almack et al., 2023; Leonard et al., 2022; Michie et al., 2018). This will have the dual outcome of showcasing great science and providing role models for Indigenous students, who are underrepresented in science (Cronin et al., 2021). Our goal is not to separate *Indigenous Science and Practice* from other contributions to *Ecology and Evolution*, but to shine a light on examples of this work so that it becomes an integrated practice in the diverse array of scientific approaches (Leonard et al., 2022). Indigenous science may be fundamentally different from non‐Indigenous (‘Western’) science, based on relational accountability (Wilson, 2008), with research interconnected to the community and driven by relationships with the community (Reid et al., 2024). We hope this special issue will provide a pathway to educate non‐Indigenous scientists on these powerful approaches to delivering insights into fundamental questions in ecology and evolution.

An important aspect of this special issue is to provide space for a wide variety of contributions which may vary in style, format, methods or goals (Wilson et al., 2020). Research conducted using Indigenous methods, questions, reporting, and with cultural and community context will be welcomed. We recognize that many Indigenous scholars are operating in a Western science‐dominated field, but that Indigenous identity can also bring valuable perspectives to questions addressed with primarily Western scientific methods. A recent study showed that increasing the proportion of female researchers led to advances in the understanding of female birdsong (Haines et al., 2020); we anticipate that increasing the representation of Indigenous first authors will also lead to new insights, questions and research directions.

We also welcome researchers who work respectfully with Indigenous knowledge holders and communities (sensu Loseto et al., 2020; Reid et al., 2024), and those who apply a two‐eyed seeing lens in their research approach (e.g. Reid et al., 2021; Service et al., 2014). We expect that research papers led by non‐Indigenous partners would be sympathetic, respectful and ethical (Louis, 2007; Wilson et al., 2020), would exceed basic standards of community consent and data ownership, and adhere to the United Nations Declaration on the Rights of Indigenous Peoples (UNDRIP). Projects that are co‐created with communities would be especially welcomed, as will papers delineating and showcasing methodologies and processes. We support the inclusion of Indigenous knowledge holders as study co‐authors when traditional ecological/local ecological knowledge is shared (Cooke et al., 2021).

Editors for this special section will include Indigenous scholars and scientists with expertise in working in partnership with Indigenous communities. These dedicated editors will handle manuscripts submitted through this call for papers, ensuring that submissions are assessed through a context‐sensitive lens. At Ecology and Evolution, our goal is to be author‐friendly (https://onlinelibrary.wiley.com/doi/10.1002/ece3.5) and ensure our reviewers are open to papers that vary in approach, method and style. In 2019, a workshop was held at the ArcticNet annual conference to discuss strategies to include Indigenous science, knowledges and researchers in the peer‐review process. Among many topics discussed, attendees spoke on the ‘importance of validating research findings, particularly those involving Indigenous Peoples and their knowledge, with the appropriate people and knowledge holders, especially Indigenous Knowledge holders’ (Loseto et al., 2020). If you are interested in being a part of the reviewing and editing process, please contact us. We wish to grow and support a broad community of reviewers who understand and appreciate Indigenous science and practice as it applies to ecology and evolution.

We look forward to receiving submissions for this special issue and expect that through these manuscripts we will continue to learn how to best support and promote Indigenous science and scientists in publishing, and support communities in delivering insights into key scientific questions and global challenges. We encourage questions, inquiries and advice in this process.

## POSITIONALITY STATEMENTS

Emily A. McKinnon is a white settler living on Treaty 1 territory in Winnipeg, Manitoba, ancestral lands of the Anishinaabeg, Ininiwak, Anisininewuk, Dakota Oyate, Dene and the homeland of the Red River Métis, with roots in Scotland, and Mi'kma'ki (Nova Scotia, Canada). Emily's work supporting Indigenous undergraduate students in science has led her to emphasize Indigenous science approaches and scientists in her teaching, and apply principles of inclusive pedagogy in the classroom. Emily is currently working on involving Indigenous students and communities in research on how climate change is impacting songbirds in Iqaluit, Nunavut and on Treaty 5 territory in Churchill, Manitoba. Emily is a senior editor for *Ecology and Evolution*.

Arley Muth is an editor‐in‐chief for *Ecology and Evolution*. Arley is a fifth‐generation White settler from the Bitterroot Valley of Montana, the ancestral lands of the Salish, Ktunaxa amakis, Cayuse, Umatilla and Walla Walla. Arley's research has focused on kelp forest ecology and climate change.

## AUTHOR CONTRIBUTIONS

**Emily A. McKinnon:** Conceptualization (equal); writing – original draft (equal). **Arley F. Muth:** Conceptualization (equal); writing – original draft (equal).

## CONFLICT OF INTEREST STATEMENT

None.

## Data Availability

No new data have been generated.
